# A Rare Cause of Severe Lower Gastrointestinal Bleeding in Ulcerative Colitis Patients in Remission: Giant Pseudopolyps

**DOI:** 10.5152/tjg.2024.24274

**Published:** 2024-10-07

**Authors:** Ali Atay, Ilhami Yuksel

**Affiliations:** 1 Department of Gastroenterology, Ankara Bilkent City Hospital, Ankara, Türkiye; 2 Department of Gastroenterology, Ankara Yıldırım Beyazıt University School of Medicine, Ankara, Türkiye

Dear Editor,

Pseudopolyps result from inflammation and regeneration cycles and may signify past severe colitis attacks. Their incidence increases in extensive colitis, affecting 10%-20% of patients with inflammatory bowel disease.^1^ When exceeding 1.5 cm in diameter, they are termed giant pseudopolyps (GPP).^2^ Although complications are rare, symptoms such as weight loss, rectal bleeding, diarrhea, abdominal pain, and obstruction may occur.^3^

A 51-year-old man was admitted to the emergency department with sudden-onset rectal bleeding occurring 10 times a day for 5 days. His medical history included extensive ulcerative colitis (UC) diagnosed 4 years earlier, which had been well controlled with azathioprine, mesalamine tablets, and enemas. However, colonoscopy revealed small and large pseudopolyps in the transverse and descending colon. Colonoscopy performed 3 months before emergency department presentation revealed GPP, but no endoscopic evidence of active disease within the mucosal area surrounding the pseudopolyps or in the rectum.

Laboratory assessments upon emergency department admission revealed the following: Hb: 5.7 g/dL, Hct: 19.9%, MCV: 73.2 fL, ferritin: 3 μg/L, CRP: 2.29 mg/L, and sedimentation rate: 6 mm/h. Cytomegalovirus polymerase chain reaction, *Clostridium difficile* toxins, and stool microbiologic examinations were negative. Computed tomography angiography displayed diffuse wall thickening, nearly obliterating the lumen in the transverse and descending colon (white arrows in the Figure 1); nevertheless, no active bleeding or mass lesion could be found. Colonoscopic examination revealed hemorrhage from pseudopolyps (black arrows in the Figure 1), while there was mild inflammation on the mucosa in the rectum and throughout the colon between pseudopolyps, indicating endoscopic remission (endoscopic Mayo score: 1, black asterisks in the Figure 1). Thus, subtotal colectomy with ileo-rectal anastomosis was performed without medical treatment escalation. In the microscopic examination of the operation material, there were cryptitis, crypt abscess, crypt distortion, and intense hemorrhage in the lamina propria (black and white asterisk and arrows in the Figure 1), but no dysplasia-associated mass or lesion was detected. The patient has remained in remission with rectal mesalamine for the past 6 months after surgery, and colonoscopy revealed normal rectal mucosa and ileo-rectal anastomosis.

Literature reported complications of GPP, with 19% experiencing obstruction, 4% intussusception, and less frequently, bloating, diarrhea, abdominal pain, protein-losing enteropathy, anemia, and melena.^4^ Additionally, reported data emphasize a higher association of pseudopolyps with increased need for biological therapy and surgery.^2,5^ While argon plasma coagulation, endoscopic loop polypectomy, and laser have been reported to be beneficial in bleeding caused by pseudopolyps.^2^ In our patient, escalation of medical treatment was not considered as the disease was graded endoscopically as mild. Additionally, the widespread bleeding foci in both giant and smaller pseudopolyps made it challenging to focus on a specific area during treatment. As a result, it was concluded that endoscopic local treatment methods would likely fail.

We present a unique case of a patient with GPP experiencing severe lower gastrointestinal bleeding due to GPP. In UC patients in remission, severe lower intestinal bleeding from pseudopolyps should be kept in mind.

## Availability of Data and Materials:

The data that support the findings of this study are available on request from the corresponding author.

## Figures and Tables

**Figure 1.**
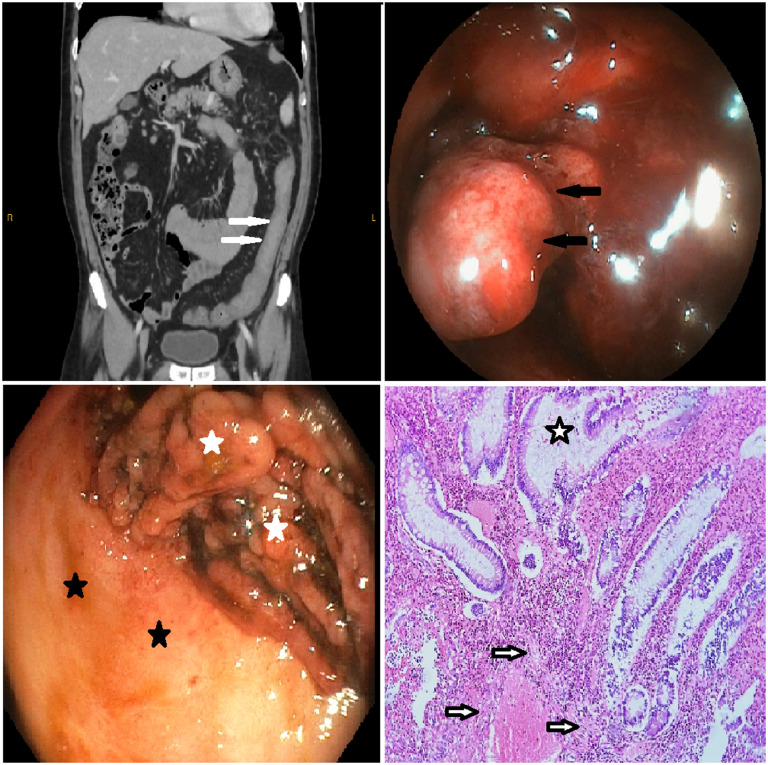
Diffuse wall thickening, nearly obliterating the lumen in the descending colon on the computed tomography (white arrows); bleeding from a giant pseudopolyp (black arrows), mildly inflamed mucosa (black asterisk) near the giant pseudopolyp (white asterisks) in the rectum in endoscopic evaluation; and crypt distortion (black and white asterisks) with erythrocyte and leukocyte accumulations around fibrin clusters formed due to hemorrhage (black and white asterisks) at microscopic examination.
